# OLDER LESBIAN HEALTH VIA RELATIONSHIP STATUS: HEALTH CONDITIONS AND HEALTH BEHAVIORS

**DOI:** 10.1093/geroni/igz038.1106

**Published:** 2019-11-08

**Authors:** Jie Yang, Paige Averett

**Affiliations:** 1 East Carolina University School of Social Work, Greenville, North Carolina, United States; 2 East Carolina University, Greenville, North Carolina, United States

## Abstract

Existing research has demonstrated that those in committed relationships are healthier than those who are not. However, very little research on same-sex relationships and particularly older lesbian relationships exists. The current study fills the gap by providing a health profile of lesbians in different relationship status, including partnered, widowed, casual dating, single—not dating, and celibate. The outcomes include diagnosed health conditions (include arthritis, high blood pressure, diabetes, depression, heart problems, and stroke) and health behaviors (exercise, smoking, and drinking). Data are from an original sample of 456 older lesbians aged 55 and over. Convenience sampling was adopted for this hard-to-reach population. Bivariate analysis results showed that widowed and celibate lesbians are more likely to report depression than other relationship groups. No disparity was found regarding health behaviors among different relationship groups. To further understand the unique group identifying as celibate, multivariate logistic regression analysis was performed to examine celibacy’s impact on lesbians’ reported depression after controlling for demographics and reported physical health. Multiple imputations was performed to handle missing values. Results showed that being celibate was associated with higher odds of reporting depression compared with having a partner or casually dating. Being celibate was also associated with lower odds of reporting depression compared with widows and singles who are not dating. To our knowledge, this study is the first to examine in details the health consequence of different relationship status among older American lesbians. Findings have important implication for health promotion among older lesbians.

